# Intravenous Single-Dose Toxicity of Redaporfin-Based Photodynamic Therapy in Rodents

**DOI:** 10.3390/ijms161226162

**Published:** 2015-12-08

**Authors:** Luis B. Rocha, Fábio Schaberle, Janusz M. Dąbrowski, Sérgio Simões, Luis G. Arnaut

**Affiliations:** 1 Luzitin SA, S. Martinho do Bispo, Coimbra 3045-016, Portugal; lrocha@luzitin.pt (L.B.R.); fschaberle@luzitin.pt (F.S.); 2 Bluepharma—Indústria Farmacêutica SA, S. Martinho do Bispo, Coimbra 3045-016, Portugal; ssimoes@bluepharma.pt; 3 Faculty of Chemistry, Jagiellonian University, Krakow 30-060, Poland; jdabrows@chemia.uj.edu.pl; 4 Chemistry Department, University of Coimbra, Coimbra 3004-535, Portugal

**Keywords:** photodynamic therapy, cancer treatment, bacteriochlorin, redaporfin, intravenous formulation, single-dose toxicity, rodents

## Abstract

We assessed the tolerability and safety in rodents of a single intravenous (i.v.) dose of redaporfin, a novel photosensitizer for Photodynamic Therapy (PDT) of cancer. Two approaches were used to evaluate acute toxicity: (i) a dose escalation study in BALB/c mice to evaluate the maximum tolerated dose of redaporfin; and (ii) a safety toxicology study in Wistar rats, of a single dose of redaporfin, with or without illumination, to evaluate possible signs of systemic toxicity. Redaporfin formulation was well tolerated by mice, with no signs of adverse reactions up to 75 mg/kg. In rats, there were no relevant changes, except for a significant, but transient, increase in the blood serum markers for hepatic function and muscle integrity, and also on neutrophil counts, observed after the application of light. The overall results showed that redaporfin-PDT is very well tolerated. No abnormalities were observed, including reactions at the injection site or skin phototoxicity, although the animals were maintained in normal indoor lighting. Redaporfin also showed a high efficacy in the treatment of male BALB/c mice with subcutaneously implanted colon (CT26) tumours. Vascular-PDT with 1.5 mg/kg redaporfin and a light dose of 74 J/cm^2^ led to the complete tumour regression in 83% of the mice.

## 1. Introduction

Photodynamic Therapy (PDT) is generally recognized as a safe and effective strategy and is approved to treat several forms of cancer. PDT requires the simultaneous combination of a drug, molecular oxygen, and light of a specific wavelength to generate reactive oxygen species (ROS), which are responsible for the destruction of tumour cells [1,2]. The antitumour effect of PDT is related to three distinct effects responsible for tumour cell death: oxidative damage promoted by ROS leading to apoptosis, autophagy or necrosis; shutdown of the tumour blood vessels that interrupts oxygen and nutrient supply; and eventually an antitumor immune response induced by PDT [3,4]. A most often presented advantage of PDT over other cancer therapies is the use of drugs—called photosensitizers (PS)—that are inactive in the absence of light. It is expected that the PS used in PDT are not toxic, or immunogenic, before they are activated by light of a wavelength specifically absorbed by the PS. When the PS is administered with an injectable solvent, the concerns on the toxicity extend to the drug formulation. The toxicity of PS and drug formulation must be assessed before entering clinical trials.

Porfimer sodium (Photofrin^®^) is currently the most used photosensitizer for systemic administration. It is clinically employed for the treatment of lung, oesophagus, bile duct, bladder, brain and ovarian tumours. Temoporfin (Foscan^®^) is a photosensitizer clinically applied in Europe for the treatment of head & neck, lung, brain, skin and bile duct cancer. Verteporfin (Visudyne^®^) met with considerable success as a photosensitizer in the treatment of age-related macular degeneration (AMD), and is also applied in PDT of prostate and pancreatic cancers. Every new drug candidate must be subjected to extensive nonclinical toxicology programs to demonstrate that it has acceptable profiles of tolerability and safety, and guarantee the lowest possible level of risk for the participants in the first-in-human trial. The general requirements for these toxicology studies are described in the harmonized guideline ICH M3(R2), from the International Council for Harmonisation of Technical Requirements for Pharmaceuticals for Human Use, and are further detailed in the comprehensive set of guidelines related to safety (S guidelines) [5]. PS for PDT may elicit intrinsic adverse reactions, may employ drug formulations with some toxicity, or may show toxicity as a consequence of the illumination protocol. This last concern is specific of PDT and requires the design of safety evaluation strategies that take into consideration the combination of drug and light [6,7].

We have been involved in the development of a fluorinated sulphonamide bacteriochlorin named redaporfin—5,10,15,20-tetrakis (2,6-difluoro-3-*N*-methylsulfamoylphenyl) bacteriochlorin, code name LUZ11—for PDT of cancer. Redaporfin presents near-ideal properties [8] and proved to be highly effective in the treatment of mice with subcutaneous colon CT26 tumour, with significant antitumor effects on distant metastasis, attributed to its ability to activate the host immune system [9,10]. Redaporfin is amphiphilic with an *n*-octanol: water partition coefficient of 80 (log*P*_OW_ = 1.9) [8]. Redaporfin is not soluble in water and tends to aggregate when a stock solution of redaporfin in an organic solvent (e.g., ethanol or dimethylsulfoxide) is added to water. In order to avoid aggregation, the redaporfin formulation contains a small amount of Cremophor EL^®^ (CrEL). CrEL has been extensively used and characterized to deliver drugs with log *P*_OW_ > 1. CrEL forms micelles in aqueous solutions and its critical micellar concentration is 0.009% (weight/volume) in protein-free aqueous solution [11]. It was noted that CrEL concentrations >0.03% in human serum lead to lipoprotein degradation [12]. Hypersensitivity reactions of drugs formulated with CrEL have been associated with concentrations >0.2% in plasma of cancer patients [13], but the adverse effects can be countered by pre-medication. Considering that the human blood plasma volume is 35 mL/kg, a total safe dose of CrEL should be <0.07 mL/kg. However, the low solubility of very lipophilic drugs may require an increase in the CrEL content in the drug formulation, which then requires pre-medication to control adverse effects. For example, the paclitaxel formulation is associated with the administration of 0.37 mL/kg of CrEL [14], but hypersensitivity reactions can be avoided with, for example, pre-medication with dexamethasone [15]. The minimum value of CrEL may alternatively be limited by its critical micellar concentration (CMC > 0.003 mL/kg). As will be shown below, our toxicology study employed a redaporfin formulation in CrEL/EtOH/NaCl 0.9% (1.2:5.7:93.1, *v*:*v*:*v*) with a redaporfin concentration of 0.86 mg/mL to administer a redaporfin dose of 1.5 mg/kg, *i.e.*, a CrEL dose of 0.021 mL/kg. This is above the CMC and well within the limits of the safe dose level. On the other hand, the dose escalation study explored redaporfin doses up to 75 mg/kg and the CrEL content of the formulation used in this study had to be increased to the proportion CrEL/EtOH/NaCl 0.9% (5:10:85, *v*:*v*:*v*), leading to the a total CrEL dose of 0.5 mL/kg.

European Medicines Agency (EMA) recently granted the status of orphan drug designation for redaporfin-PDT of biliary track cancer [16], and redaporfin entered clinical trials with patients with advanced head and neck cancer [17]. This work reports preliminary safety studies designed to assess the safety and tolerability of redaporfin-PDT in the clinical trial application of redaporfin. The studies cover a dose escalation study in mice to evaluate the Maximum Tolerated Dose (MTD) of redaporfin in its formulation but without illumination, and a systemic toxicity study in rats, with and without illumination. Since the goal is for the redaporfin-PDT in the clinic to be effective with only one treatment, both studies focused on the acute reactions elicited by a single session of PDT. This work also presents an efficacy study designed to test a 1.5 mg/kg dose of redaporfin with a light dose of 74 J/cm^2^ in vascular-PDT, with a drug-light interval (DLI) of 15 min. These doses are higher than those used in previous studies with redaporfin [9,10], and motivated the use of male, rather than female, BALB/c mice in this study, to take advantage of their larger size (25 g *vs.* 20 g). The use of higher doses in this study explores the relation between the onset of adverse effects and the size of the animal-models.

## 2. Results

### 2.1. Dose Escalation Study

This study consisted in the i.v. administration of increasing concentrations of redaporfin formulation to female BALB/c mice, without illumination. 

Throughout the study, there were no noticeable changes in the overall condition of the mice. No significant variations were observed in the average body weights (BW) in any of the study groups, including the control group that received the vehicle alone. The BW evolution over time after the administration of redaporfin for each group is presented in Table 1. The eyes, tail and paws of the animals were observed regularly and did not indicate signs of photosensitivity reactions due to exposure to indoor lighting. In addition, mice behaviour was consistent throughout the study, without signs of light avoidance during the handling procedures. Moreover, there were no signs of local reaction at the site of injection. 

Mice from group G6 (75 mg/kg) started the study eight weeks after the other groups, once it was concluded that the injected redaporfin formulations up to 37.5 mg/kg did not cause any observable reaction in mice. This time gap accounts for the higher average body weight observed in G6 group as compared to the other groups.

### 2.2. Safety Toxicology Study

In this study, female Wistar rats received an i.v. injection of redaporfin formulation, followed or not, by laser illumination to evaluate possible signs of systemic toxicity, through standard clinical blood analysis.

All animals enrolled in this study survived until the time-points defined for terminal blood collection, without any observable signs of adverse reactions that could be associated with the i.v. administration of the redaporfin formulation, the vehicle alone or the PDT protocol. Changes in the general condition or normal behaviour of the animals were not detected, even in the group that received 15 mg/kg or in the irradiated groups. There was no sign of skin phototoxicity reactions when they were exposed to the standard laboratory illumination, which indicates the absence of light sensitivity under those conditions.

Three study groups were irradiated in the right thigh with a DLI of 15 min, meaning that the rats were irradiated 15 min after the administration of redaporfin. On the following day, 24 h after illumination, the animals presented a significant inflammatory response in the irradiated leg, indicated by a large oedema, although it did not interfere with their movements. In addition, a necrotic eschar covering the irradiated area was visible 72 h after PDT in all animals from the irradiated groups. Possible signs of systemic toxicity were accessed by a standard set of clinical blood tests at selected time-points, post-administration/illumination. Such tests are routinely performed to evaluate acute and chronic toxicity reactions through the measurement of haematological parameters and biochemistry markers, and are very useful in the non-clinical evaluation of the safety profile of new drug candidates, including PS [18,19]. Generally, they provide relevant information about the circulatory homeostasis, liver and renal function or muscle injury [20]. 

**Table 1:** Mice body weight (g) over time after the i.v. injection of redaporfin formulation for each study group (average ± SD).

Group	Redaporfin (mg/kg)	Days After Administration									
		0	4	7	11	14	16	21	28	35	46
G0	0	21.9 ± 0.6	-	21.9 ± 0.8	-	-	22.2 ± 1.0	-	22.1 ± 1.2	22.4 ± 0.8	-
G1	15	22.0 ± 0.7	-	21.8 ± 0.6	-	-	22.4 ± 0.6	-	22.9 ± 0.5	23.1 ± 0.6	-
G2	21	22.0 ± 1.0	-	21.7 ± 0.8	-	-	22.1 ± 0.9	-	22.3 ± 1.0	22.1 ± 1.1	-
G3	26.3	21.4 ± 0.5	-	20.8 ± 0.7	-	-	21.0 ± 0.7	-	21.9 ± 1.1	21.7 ± 0.4	-
G4	30	22.8 ± 0.9	-	22.5 ± 0.9	-	-	22.6 ± 1.0	-	22.3 ± 0.9	23.4 ± 1.0	-
G5	37.5	21.9 ± 1.2	-	21.5 ± 1.0	-	-	21.7 ± 1.0	-	21.8 ± 1.3	22.2 ± 1.0	-
G6	75	24.4 ± 1.7	23.9 ± 1.6	24.2 ± 1.7	24.2 ± 1.6	24.2 ± 1.6	-	24.5 ± 1.8	25.5 ± 1.6	-	25.3 ± 1.7

The detailed results of the blood tests performed for all study groups are presented in Appendix A (Table A1—haematology and Table A2—serum biochemistry). The values determined for the non-treated control group were found to be in the normal ranges for female Wistar rats with similar age, according to the supplier technical documents [21]. The haematology and serum biochemistry results obtained in the three non-irradiated groups, which received the vehicle alone, redaporfin at 1.5 or 15 mg/kg, showed no significant differences in comparison to the non-treated control group. This demonstrates that a single i.v. administration of the redaporfin formulation, even at a dose of 15 mg/kg, does not have a significant impact on the clinical blood parameters, thus showing no signs of systemic toxicity.

**Figure 1:**
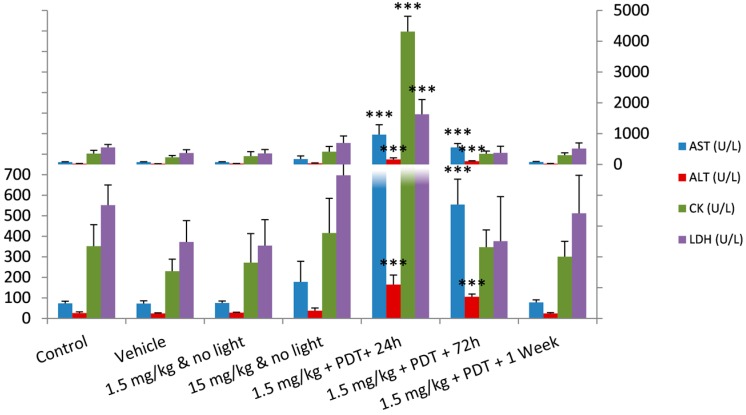
Summary of the most relevant results from serum biochemistry of Wistar rats, presented as average ± SD. (*** difference relative to the control group—*p* < 0.001). The two panels present the same results in different scales to highlight the inter-group differences.

The comparison of the serum biochemistry results between the irradiated groups and the control group revealed significant differences on the markers for liver function and muscle damage. The results show that 24 h after PDT, there was a significant increase in the levels of the hepatic transaminases, aspartate aminotransferase (AST) and alanine aminotransferase (ALT), while alkaline phosphatase (ALP) remained unchanged (Figure 1). Together with the significant rise of lactate dehydrogenase (LDH) and creatine kinase (CK), which are normally associated with muscle damage [22], these results were probably a consequence of the destruction of skeletal muscle on the irradiated thigh caused by the photodynamic effect of the redaporfin-PDT. Nevertheless, 72 h after PDT the levels of all four markers had already decreased, and only AST and ALT remained significantly higher than in the control group. One week after PDT, all the altered biochemistry parameters have returned to their pre-PDT levels, indicating that the changes in liver function were transitory and probably a consequence of muscle damage, associated with the observed local tissue destruction caused by the PDT protocol. On the other hand, there were no significant alterations on the levels of renal function, such as urea, blood urea nitrogen, creatinine or total protein, which is a good indication that the kidneys were not affected by redaporfin and its formulation, nor by the photodynamic reaction elicited by the PDT protocol. It is important to note that, in the PDT treatment of an animal with a solid tumour, the destruction of skeletal muscle would by greatly reduced, since the illumination would be directed to the tumour mass and not to the muscle, which would probably reduce the alterations in the liver function found in this study. Nevertheless, in clinical studies of redaporfin-PDT should survey markers of liver function. 

**Figure 2:**
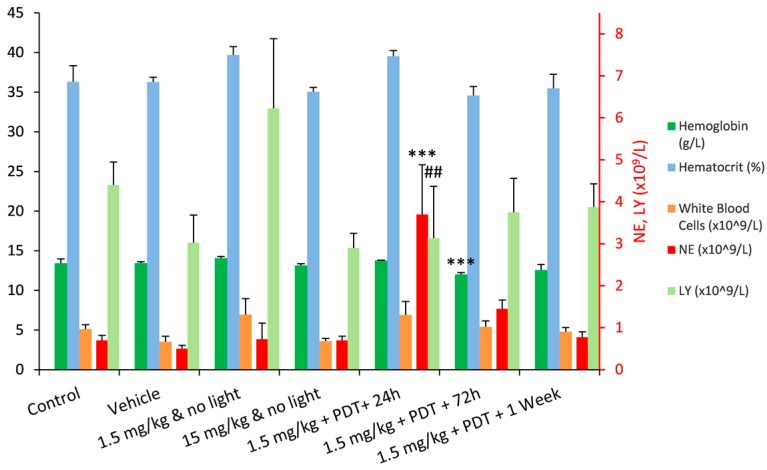
Summary of the most relevant results from Wistar rats haematology, presented as average ± SD. The values for the neutrophils (NE) and lymphocytes (LY) populations are presented in the secondary vertical axis. (*** difference relative to the control group—*p* <0.001; ^##^ difference relative to the 1.5 mg/kg group—*p* < 0.01).

The haematology parameters of all test groups were largely unaffected in comparison to the control group, with only two statistically significant exceptions: a dramatic increase in the number of circulating neutrophils observed 24 h after PDT, and a decrease in the haemoglobin level 72 h after PDT. In addition, 24 h after PDT there was a significant decrease in the lymphocyte population relative to 1.5 mg/kg group but not significant in relation to the control group (Figure 2). The increase of the neutrophil population in circulation can be associated with the innate immune system response to a local insult, which triggers an acute inflammatory response [4,23]. This response was clearly observed in the form of a large oedema that extended through the whole leg of the animals, on the days that followed the illumination. On the third day after illumination, the population of circulating neutrophils was already decreasing, which can be an indication of their passage from the circulation into the damaged tissues [24]. One week after PDT, the number of neutrophils in the blood had already returned to the levels pre-PDT.

### 2.3. Photodynamic Efficacy of Redaporfin–PDT

**Figure 3:**
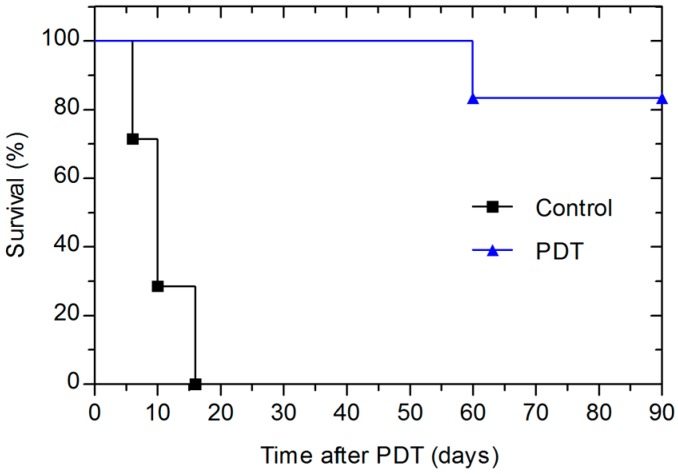
Kaplan Meier plot for BALB/c animals with CT26 untreated tumours and tumours treated with PDT with total light doses of 74 J/cm^2^ (130 mW) at 15 min after 1.5 mg/kg of redaporfin administration.

Figure 3 shows a Kaplan-Meier plot of the percentage of animals with local tumour control after one single treatment with 1.5 mg/kg redaporfin. The illumination was carried out for 9.5 min while redaporfin was confined in the vasculature (DLI = 15 min) using 130 mW laser at 749 nm. Interestingly, whereas a similar drug and light dose and the same DLI lead to overdosing in female BALB/c mice weighing approximately 20 g [10], with 3 months old male BALB/c mice weighing around 25 g this protocol did not lead to any PDT-induced lethality. Moreover, this protocol achieved a cure rate of 83%. This remarkable long-term tumour response is due to extensive necrosis of all the illuminated area. The high photodynamic activity towards CT26 tumours may be explained by efficient generation of both singlet oxygen and hydroxyl radicals by redaporfin [8,25], and the strong immune response triggered by the high local inflammation after PDT [10]. 

## 3. Discussion

The redaporfin formulations administered intravenously to mice without illumination, even at the highest dose of 75 mg/kg, seem to be very well tolerated by the animals. There were no observable signs of local irritation at the injection site, and no signs of photosensitivity appeared in any of the groups. These results are indicative of the safety of redaporfin and its formulation, confirming the very low toxicity in the absence of light observed during the *in vitro* screening stage, and suggest a comfortable safety margin in relation to the expected therapeutic dose. Indeed, the redaporfin-PDT protocol with DLI = 15 min, 1.5 mg/kg redaporfin and a light dose of 74 J/cm^2^, proved to be effective and safe for male BALC/c mice. This means that the formulation used in the clinical studies, CrEL/EtOH/NaCl 0.9% (0.2:1:98.8, *v*:*v*:*v*), may deliver an effective drug dose at a CrEL level close to the CMC limit and well below the safe dose limit.

Safety toxicology in rats followed clinical blood-markers at different time-points after i.v. injection of redaporfin and after PDT to evaluate possible systemic toxicity. There were no visible signs of irritation at the injection site, and no light-avoidance behaviour. The blood markers confirmed that the redaporfin formulation, either at 1.5 mg/kg or at 15 mg/kg, without the application of a PDT protocol is very well tolerated and does not cause significant alterations on the baseline levels of the evaluated parameters. The scenario was different when a vascular-PDT protocol was applied in healthy rats. This protocol used 1.5 mg/kg of redaporfin, a DLI of 15 min and a light fluence of 74 J/cm^2^, which was shown to be very efficient in the PDT treatment of BALB/c male mice (83% cures). In healthy rats, it led to a strong local inflammatory response in and around the illuminated area, and to the formation of a necrotic scab covering perfectly the illuminated tissue. In addition to these macroscopic observations, some of the clinical markers for the hepatic function and muscle integrity showed a large increase 24 h after PDT, which are assigned to the considerable destruction of skeletal muscle caused by redaporfin-PDT in this model. On the day after PDT, there was also a significant but transient increase of the population of neutrophils in circulation, which could be a result of their recruitment in response to the local inflammatory reaction to infiltrate the affected tissues. Nevertheless, these acute changes were temporary, as demonstrated by their attenuation 72 h after the illumination and after one week returned to their levels before PDT. The reactions to the i.v. administration of redaporfin are completely reversible and far less significant than those reported in the scientific discussion document of the EMA for Foscan^®^, which was approved as systemic photosensitizer for head and neck cancer. The i.v. administration of Foscan^®^ to mice and rats exposed only normal light conditions, without illumination, led to phototoxicity reactions on exposed areas of the skin and to systemic toxicity characterized by significant changes in haematological and haematopoietic parameters and increased spleen and liver weights, even at doses as low as 0.85 mg/kg [26].

It is interesting to remark that the vascular-PDT protocol used in this study with male BALB/c mice (1.5 mg/kg and 98 J) did not lead to PDT-induced lethality, whereas a similar protocol with female BALB/c mice (1.5 mg/kg and 78 J) led to the death of five out of six the animals in less than three days after PDT [9,10]. We assign this difference to the smaller size of the female BALB/c mice, which had between 16.9 g and 19.4 g at the time of that treatment, with respect to the male BALB/c mice in this study, which weighed approximately 30 g. PDT-induced lethality decreases dramatically with body weight for the same drug and light dose when the light dose is expressed in Joules, or in J/cm^2^ and the illuminated area is kept constant. The tissue damage produced by the ROS does not scale with the body weight for a constant light dose. For example, a 20 g mouse has a surface area of 66 cm^2^ and we illuminated 1.33 cm^2^ [27]. A 60 kg adult has a surface area of 1.6 m^2^ and the equivalent PDT-induced lethality dose must take in account both the weight of the patient to establish the drug dose and the surface area illuminated to establish the light dose.

## 4. Materials and Methods

### 4.1. Chemicals

The redaporfin was supplied by Luzitin, SA in sealed vials with weighed amounts under nitrogen atmosphere, and was stored at approximately −18 °C, in the dark. All procedures involving the handling of redaporfin, either as a solid or in solution, were performed in conditions of reduced luminosity (in the absence of direct light). Cremophor^®^ EL was purchased from Sigma-Aldrich (St. Louis, MO, USA). Absolute ethanol and NaCl were obtained from Merck (Darmstadt, Germany).

### 4.2. Animals

The use of laboratory animals in this study was authorized by the Portuguese Veterinary Authority—authorization number 0420/000/000/2011. BALB/c female mice with 8 weeks of age and female Wistar Han rats with 10 weeks of age were supplied by Charles River Laboratories (Barcelona, Spain). They were maintained with free access to food and water in a room with controlled cycle of 12 h light/dark. At the end of the study, animals were anaesthetised with a mixture of ketamine 100 mg/kg (Clorketam 1000, Vetoquinol, Barcarena, Portugal) and xylazine 10 mg/kg (Rompun 2%, Bayer, Carnaxide, Portugal) and sacrificed by cervical dislocation.

### 4.3. Dose Escalation Study

#### 4.3.1. Redaporfin Formulations

Each test dose of redaporfin (15, 21, 26.3, 30, 37.5 and 75 mg/kg) was prepared as an individual formulation for i.v. administration. The formulation containing CrEL/EtOH/NaCl 0.9% was the same for all doses and was a modified version of the formulation developed and optimized for redaporfin-PDT of BALB/c [9,10], and DBA/2 mice [25] with subcutaneously implanted colon CT26 and melanoma S91 tumours, respectively. The proportions of CrEL and EtOH relative to redaporfin had to be significantly increased in this study to allow for the complete solubilisation of the necessary amount of redaporfin to reach the defined maximum dose of 75 mg/kg. However, CrEL and EtOH were kept within the recommended limits for i.v. administration in mice [28,29]. The modified formulation CrEL/EtOH/NaCl 0.9% (5:10:85, *v*:*v*:*v*) was prepared weighing the desired amount of redaporfin was into a 2 ml microtube and dissolving it with the appropriated volumes of CrEL and absolute ethanol through alternated cycles of 30 s of vortex mixing followed by 5 min in an ultra-sound bath. Then, the solution was transferred to another tube containing the appropriated volume of NaCl 0.9% and was homogenised through vortex mixing, resulting in a limpid dark green solution. The complete solubilisation of redaporfin was confirmed by the absence of precipitate after a 5 min centrifugation at 4000 rpm. As expected, the difficulties in the solubilisation of redaporfin in the CrEL/EtOH mixture increased dramatically with the concentration of the molecule. Consequently, the formulation for 75 mg/kg of redaporfin (7.5 mg/mL) was the maximum feasible dose. 

#### 4.3.2. Intravenous Administration

The final formulations listed in Table 2 were slowly injected in the mice tail vein using a syringe with a 26G needle in a proportion of 200 µL per 20 g of mouse body weight. The first 6 groups (G0 to G5) were administered and followed in the first stage of the study. After the end of this first stage, group G6 was then administered and followed.

**Table 2:** Summary of the study groups and the correspondent administered redaporfin i.v. formulations.

Test Group	N	Redaporfin (mg/kg)	Redaporfin (mg/mL)
G0 (Control)	5	0.0	0.00
G1	5	15.0	1.50
G2	5	21.0	2.10
G3	5	26.3	2.63
G4	5	30.0	3.00
G5	5	37.5	3.75
G6	4	75.0	7.50

#### 4.3.3. Mice Follow-up

After administration, mice were evaluated at least once a week during 5 weeks. The following observations were registered: body weight, local reactions at the site of injection, light sensitivity/avoidance and general condition. 

### 4.4. Safety Toxicology Study

#### 4.4.1. Redaporfin Formulation

A redaporfin formulation in CrEL/EtOH/NaCl 0.9% was prepared first dissolving the defined amount of PS in the appropriated volumes of CrEL and absolute ethanol, using alternated cycles of 30 s of vortex mixing followed by 5 min in an ultra-sound bath. Next, the solution was transferred to a tube containing the appropriated volume of NaCl 0.9%, and was carefully homogenised, resulting in a limpid dark green solution. The complete solubilisation of redaporfin was confirmed by the absence of precipitate after a 5 min centrifugation at 4000 rpm. The final formulation for the i.v. administration of 1.5 mg/kg of redaporfin in Wistar rats was composed of CrEL/EtOH/NaCl 0.9% (1.2:5.7:93.1, *v*:*v*:*v*) and had a redaporfin concentration of 0.86 mg/mL. The formulation used to deliver 15 mg/kg of redaporfin required CrEL/EtOH/NaCl 0.9% (6.1:28.8:65.1, *v*:*v*:*v*) to allow the solubilisation of the higher amount of PS, and had a final concentration of 8.63 mg/mL of redaporfin.

#### 4.4.2. Intravenous Injection, PDT and Blood Collection

Seven groups of animals were randomly organized (*n* = 4): 4 non-illuminated groups and 3 groups that received a light dose after the administration of redaporfin. The non-illuminated were: non-treated control, redaporfin 1.5 mg/kg, redaporfin 15 mg/kg, and vehicle (the same vehicle used in the 15 mg/kg formulation, which contained the higher amounts of CrEL and EtOH). The 3 groups that received laser illumination (74 J/cm^2^, diameter of illumination Ø = 10 mm) were illuminated 15 min after the i.v. administration of redaporfin 1.5 mg/kg followed by. Rats from these 3 groups were illuminated in the muscle of the right thigh, previously shaved, using a diode laser type LA0873, S/N M070301 (Hamamatsu, Shizuoka, Japan) controlled with a ThorLabs 500 mA ACC/APC Laser Diode Controller (Munich, Germany) and in-house electronics, emitting 130 mW at 749 nm, which was hand-held during the illumination. The time-points for the terminal blood collection were 24 h, 72 h, and 1 week after PDT for the irradiated groups, and 24 h after the administration for the other groups. Due to the volume of blood needed for the haematological and biochemistry tests, the procedure for blood collection was terminal. For the administration, illumination and blood collection rats were anesthetised with an intraperitoneal injection of a mixture of ketamine 75 mg/kg and xylazine 10 mg/kg. At the defined time-points, 2–2.5 mL of blood were drawn from the abdominal aorta and, immediately after, the animals were sacrificed by cervical dislocation.

#### 4.4.3. Blood Analysis

Blood tests (haematology and serum biochemistry) were outsourced to a Clinical Analysis Laboratory and were performed using standard clinical procedures and equipment.

Immediately after collection, each blood sample was fractioned: 1 mL was transferred to an haematology tube containing ethylenediamine tetraacetic acid (EDTA) and gently homogenised, and the remaining, for serum biochemistry, was dispensed into a 2 mL microtube and was allowed to cloth at ambient temperature during 30 min. Serum was separated by centrifugation at 4000 rpm for 10 min, and 0.5 mL of supernatant were transferred to a new tube.

Blood for haematology and serum were stored at 2–8 °C and analysed in the same day of collection. For manual leukocyte differential counts, a blood smear was prepared for each blood sample after blood collection, using a drop of EDTA-anticoagulated blood from the haematology tube. When dry, the smear was fixed with methanol for 3 min, and then left to dry in vertical position. Slides were stored at ambient temperature until processing and analysis.

The haematology test evaluated the following parameters: Red Blood Cells (×10^12^/L). Reticulocytes (%). Haemoglobin (g/dL). Haematocrit (%). Mean Corpuscular Volume (fL). Mean Corpuscular Haemoglobin (pg). Mean Corpuscular Haemoglobin Concentration (g/L). Red Cell Distribution Width (%). Platelets (×10^9^/L). Mean Platelet Volume (fL). Plateletcrit (%). Platelet Distribution Width (%). White Blood Cells (×10^9^/L). White Blood Cells Differential Count.

The serum biochemistry test evaluated the following parameters: Glucose (mg/dL). Urea (mg/dL). Total Protein (g/L). Cholesterol (mg/dL). Triglycerides (mg/mL). Aspartate Aminotransferase (IU/L). Alanine Aminotransferase (IU/L). γ-Glutamyl Transpeptidase (IU/L). Alkaline Phosphatase (IU/L). Ureic Nitrogen (mg/dL). Creatine Kinase (IU/L). Lactate Dehydrogenase (IU/L). Creatinine (mg/dL). Bilirubin (mg/dL).

Results are presented for each group and time-point as average ± SD. Differences between test and control groups were evaluated by one-way analysis of variance (ANOVA), using GraphPad Prism software (V5.01), with Tukey’s *post hoc* test for multiple pair-wise comparisons. Differences were considered statistically significant for *p* < 0.05.

### 4.5. PDT Treatment 

Male BALB/c mice, approximately 3 months old (*ca.* 25 g), were implanted with 500,000 CT26 cells in the left thigh, and the tumours were allowed to grow to a volume of 30–50 mm^3^. Redaporfin formulation were prepared by dissolving the desired amount of redaporfin in a given volume of CrEL (Sigma, Steinheim, Germany) mixed in 1:1 (*v*:*v*) proportion with ethanol 99.8% (Sigma) followed by dilution by a factor of 50 in NaCl 0.9% aqueous solution. When the tumours reached the size for the treatment, an appropriate volume of a freshly prepared redaporfin formulation was i.v. injected to administer at a dose of 1.5 mg/kg body weight. Illumination of the tumour area was performed with an Omicron laser model LDM750.300.CWA.L.M (Rodgau-Dudenhofen, Germany), emitting at 749 nm, connected to a glass optical fibre model FD (Medlight, Ecublens, Switzerland) coupled with a microlens, which was held in a fixed position and directed to the tumour. The protocol parameters varied in this study were: (i) DLI = 15 min; (ii) redaporfin dose = 1.5 mg/kg; (iii) light dose = 74 J/cm^2^; (iv) laser output power = 130 mW; (v) Ø = 13 mm.

## 5. Conclusions

The exploratory dose escalation and toxicology studies presented in this work demonstrate the low toxicity of redaporfin and its formulation, even when combined with a biologically effective PDT protocol. The only trace of toxicity was the observation of elevated transaminases after illumination of healthy tissue. These studies were confirmed and complemented with pharmacology and toxicology studies in rat and dog, performed by a contract research organization (CRO) in accordance to all regulatory good laboratory practice (GLP) requisites, to complete the nonclinical package required for the clinical trial application for advanced cancer. Nevertheless, the demonstration that our redaporfin formulations are well-tolerated and non-toxic to rodents, does not totally remove the possibility of observing hypersensitivity reactions in humans, because rodents are much less sensitive to Cremophor EL than humans. The introduction in the clinic of formulations containing CrEL must always be accompanied by careful surveillance of hypersensitivity reactions. Remarkably, redaporfin-PDT of a BALB/c mouse model with a drug dose 50 times lower than the highest dose tested in the toxicity study, provided significant survival advantage, with a cure rate of 83%. This is an encouraging result for one single PDT treatment of subcutaneously implanted CT26 colon carcinoma.

## Abbreviations

The following abbreviations are used in this manuscript: γ-GT: Gamma-glutamyl transferase; ALP: alkaline phosphatase; ANOVA: analysis of variance; ALT: alanine aminotransferase; AST: aspartate aminotransferase; BA: basophils; BIL: total bilirubin; BUN: blood urea nitrogen; BW: body weight; CHOL: total cholesterol; CK: creatine kinase; CRE: creatinine; CrEL: Cremophor EL; CRO: Contract Research Organization; DLI: drug-light interval; EDTA: ethylenediamine tetraacetic acid; EMA: European Medicines Agency; EO: eosinophils; EtOH: ethanol; GLP: Good Laboratory Practice; GLU: glucose; Hb: haemoglobin; HCT: haematocrit; ICH: International Council for Harmonisation of Technical Requirements for Pharmaceuticals for Human Use; i.v.: intravenous; LDH: lactate dehydrogenase; LY: lymphocytes; MCH: mean corpuscular haemoglobin; MCHC: mean corpuscular haemoglobin concentration; MCV: mean corpuscular volume; min: minutes; MO: monocytes; MPV: mean platelet volume; MTD: maximum tolerated dose; NE: neutrophils; PCT: plateletcrit; PDT: Photodynamic Therapy; PDW: platelet distribution width; PLT: platelets; PS: photosensitizer; RBC: red blood cells; RDW: red blood cell distribution width; Retic: reticulocytes; ROS: reactive oxygen species; SD: standard deviation; TG: triglycerides; TP: total protein; WBC: white blood cells.

## Appendix A

**Table A1:** Results of the haematology tests from all study groups, presented as average ± SD.

Haematology	Control	Vehicle	1.5 mg/kg	15 mg/kg	1.5 mg/kg PDT + 24 h	1.5 mg/kg PDT + 72 h	1.5 mg/kg PDT + 1 week
RBC (×10^12^/L)	6.5 ± 0.3	6.5 ± 0.3	7.2 ± 0.3	6.4 ± 0.2	6.7 ± 0.3	6.1 ± 0.2	6.2 ± 0.4
Retic (%)	1.3 ± 0.1	1.4 ± 0.1	1.3 ± 0.1	1.4 ± 0.1	1.2 ± 0.1	1.3 ± 0.1	NP
Hg (g/L)	13.4 ± 0.5	13.5 ± 0.2	14.1 ± 0.2	13.1 ± 0.2	13.8 ± 0.1	12.0 ± 0.2	12.6 ± 0.7
HCT (%)	36.3 ± 2.0	36.3 ± 0.6	39.7 ± 1.0	35.1 ± 0.6	39.5 ± 0.7	34.6 ± 1.1	35.5 ± 1.8
MCV (fL)	56.1 ± 1.7	55.1 ± 1.8	55.2 ± 1.3	55.0 ± 1.3	59.5 ± 2.0	56.9 ± 1.8	57.1 ± 1.0
MCH (pg)	20.8 ± 0.8	20.6 ± 1.1	19.6 ± 0.9	20.6 ± 0.7	20.7 ± 1.0	19.8 ± 0.5	20.2 ± 0.3
MCHC (g/L)	37.1 ± 0.8	37.3 ± 0.8	35.5 ± 0.8	37.4 ± 0.5	34.9 ± 0.5	34.8 ± 0.6	35.4 ± 0.6
RDW (%)	12.0 ± 0.4	12.3 ± 1.1	12.3 ± 1.3	12.7 ± 1.0	11.2 ± 0.6	11.9 ± 0.9	13.0 ± 1.3
PLT (x10^9^/L)	740 ± 62	629 ± 90	801 ± 56	654 ± 83	615 ± 167	610 ± 34	NM
MPV (fL)	5.8 ± 0.2	5.6 ± 0.3	5.4 ± 0.1	5.7 ± 0.2	6.1 ± 0.4	5.7 ± 0.2	5.7 ± 0.2
PCT (%)	42.7 ± 2.4	35.0 ± 3.4	43.4 ± 2.4	37.4 ± 6.1	37.4 ± 8.6	35.0 ± 1.0	67.3 ± 9.4
PDW (%)	17.0 ± 0.2	16.7 ± 0.6	16.9 ± 0.8	16.8 ± 0.5	17.2 ± 0.4	16.7 ± 0.3	16.6 ± 0.3
WBC (×10^9^/L)	5.1 ± 0.6	3.5 ± 0.7	7.0 ± 2.0	3.6 ± 0.4	6.9 ± 1.7	5.4 ± 0.8	4.8 ± 0.5
NE (%)	16.0 ± 6.4	14.0 ± 3.5	11.0 ± 5.0	20.7 ± 9.3	53.3 ± 10.2	28.3 ± 7.8	NP
NE (×10^9^/L)	0.8 ± 0.2	0.5 ± 0.1	0.8 ± 0.5	0.8 ± 0.4	3.7 ± 1.0	1.5 ± 0.4	NP
LY (%)	82.0 ± 6.3	84.3 ± 3.4	85.3 ± 5.0	76.0 ± 9.5	41.3 ± 10.0	66.0 ± 7.5	NP
LY (×10^9^/L)	4.2 ± 0.7	3.0 ± 0.6	5.9 ± 1.6	2.7 ± 0.2	2.9 ± 1.2	3.6 ± 0.7	NP
MO (%)	1.8 ± 1.5	1.8 ± 2.1	3.0 ± 0.8	3.3 ± 1.5	5.3 ± 0.6	5.0 ± 2.2	NP
MO (×10^9^/L)	0.1 ± 0.1	0.1 ± 0.1	0.2 ± 0.1	0.1 ± 0.1	0.4 ± 0.1	0.3 ± 0.1	NP
EO (%)	0.3 ± 0.5	0.0 ± 0.0	0.8 ± 1.0	0.0 ± 0.0	0.0 ± 0.0	0.8 ± 1.0	NP
EO (×10^9^/L)	0.0 ± 0.0	0.0 ± 0.0	0.1 ± 0.1	0.0 ± 0.0	0.0 ± 0.0	0.0 ± 0.1	NP
BA (%)	0.0 ± 0.0	0.0 ± 0.0	0.0 ± 0.0	0.0 ± 0.0	0.0 ± 0.0	0.0 ± 0.0	NP
BA (×10^9^/L)	0.0 ± 0.0	0.0 ± 0.0	0.0 ± 0.0	0.0 ± 0.0	0.0 ± 0.0	0.0 ± 0.0	NP

NP = Not performed due to sample degradation during blood smear processing; NM = Not measurable – out of range; BA: basophils; EO: eosinophils; HCT: haematocrit; Hg: haemoglobin; LY: lymphocytes; MCH: mean corpuscular haemoglobin; MCHC: mean corpuscular haemoglobin concentration; MCV: mean corpuscular volume; MO: monocytes; MPV: mean platelet volume; NE: neutrophils; PCT: plateletcrit; PDW: platelet distribution width; PLT: platelets; PS: photosensitizer; RBC: red blood cells; RDW: red blood cell distribution width; Retic: reticulocytes; WBC: white blood cells.

**Table A2:** Results of the serum biochemistry tests from all study groups, presented as average ± SD.

Biochemistry	Control	Vehicle	1.5 mg/kg	15 mg/kg	1.5 mg/kg PDT + 24 h	1.5 mg/kg PDT + 72 h	1.5 mg/kg PDT + 1 week
GLU (mg/dL)	229 ± 54	218 ± 17	217 ± 46	222 ± 9	174 ± 47	171 ± 21	191 ± 34
Urea (mg/dL)	42 ± 3	33 ± 2	33 ± 4	32 ± 5	30 ± 2	40 ± 8	44 ± 2
CHOL (mg/dL)	52 ± 7	43 ± 7	51 ± 4	41 ± 7	68 ± 12	54 ± 4	55 ± 8
TG (mg/mL)	114 ± 45	84 ± 9	120 ± 62	66 ± 20	61 ± 16	58 ± 14	117 ± 53
AST (U/L)	73 ± 11	73 ± 13	76 ± 10	178 ± 100	968 ± 317	555 ± 123	78 ± 12
ALT (U/L)	27 ± 5	24 ± 3	29 ± 2	38 ± 13	165 ± 47	106 ± 13	25 ± 3
CRE (mg/dl)	0.47 ± 0.02	0.44 ± 0.03	0.45 ± 0.01	0.44 ± 0.05	0.48 ± 0.07	0.43 ± 0.02	0.49 ± 0.03
γ-GT (U/L)	NM	NM	NM	NM	NM	NM	NM
ALP (U/L)	88 ± 15	54 ± 6	93 ± 37	90 ± 12	97 ± 38	56 ± 13	60 ± 8
BIL (mg/dL)	0 ± 0	0.1 ± 0.0	0 ± 0	0.1 ± 0.1	0.1 ± 0.1	0.1 ± 0.1	0.1 ± 0.0
TP (g/L)	5.6 ± 0.2	5.2 ± 0.3	5.3 ± 0.2	5.6 ± 0.3	4.7 ± 0.3	5.3 ± 0.1	5.6 ± 0.1
CK (U/L)	352 ± 105	230 ± 58	272 ± 142	416 ± 169	4314 ± 496	347 ± 84	301 ± 74
BUN (mg/dL)	20 ± 1	15.3 ± 0.8	15 ± 2	15.1 ± 2.2	14.2 ± 1.0	18.6 ± 3.7	20.5 ± 0.7
LDH (U/L)	552 ± 97	372 ± 104	355 ± 127	698 ± 227	1625 ± 483	376 ± 217	513 ± 185

NM = Not measurable – out of range; γ-GT: Gamma-glutamyl transferase; ALP: alkaline phosphatase; ALT: alanine aminotransferase; AST: aspartate aminotransferase; BIL: total bilirubin; BUN: blood urea nitrogen; CHOL: total cholesterol; CK: creatine kinase; CRE: creatinine; GLU: glucose; LDH: lactate dehydrogenase; TG: triglycerides; TP: total protein.
